# Variation in Ovine *DGAT1* and Its Association with Carcass Muscle Traits in Southdown Sheep

**DOI:** 10.3390/genes13091670

**Published:** 2022-09-19

**Authors:** Rong Dai, Huitong Zhou, Qian Fang, Ping Zhou, Yang Yang, Shuang Jiang, Jonathan G. H. Hickford

**Affiliations:** 1 Xingjiang Academy of Agricultural and Reclamation Science/State Key Laboratory of Sheep Genetic Improvement and Healthy Production, Shihezi 832000, China; 2 Gene-Marker Laboratory, Department of Agricultural Sciences, Lincoln University, Lincoln 7647, New Zealand

**Keywords:** diacylglycerol O-acyltransferase 1 gene, polymorphism, PCR-SSCP, lamb, loin meat yield

## Abstract

Diacylglycerol O-acyltransferase 1 (DGAT1) is a microsomal enzyme that plays a key role in the synthesis of triglycerides. Its gene (*DGAT1*) is regarded as a candidate gene for variation in milk and meat traits in cattle. The objective of this study was to use a PCR single-strand conformation polymorphism approach to explore sequence variation in two regions of ovine *DGAT1* and to assess its effect on meat traits in New Zealand Southdown sheep. Three variant nucleotide sequences were identified in each region, with two single nucleotide polymorphisms (SNPs) and one nucleotide deletion being detected in intron 1 and two SNPs being found in exon 17. The effect of the exon 17 variation was not investigated due to one variant being predominant and the other two variants occurring at low frequencies. In intron 1, one variant (*B*_1_) was found to be associated with increase loin meat yield, suggesting that this may have value as a gene marker for improving meat traits.

## 1. Introduction

Fat is an unpopular constituent of meat for many consumers, as it is considered unhealthy and high intakes of saturated fat are reported to be associated with increased heart disease risks [1]. However, fat and fatty acids affect various aspects of meat quality and palatability, and they play an important role in the nutritional and market value of meat [2]. Genes regulating lipid metabolism and deposition have therefore been a research focus for the genetic improvement of livestock.

The diacylglycerol O-acyltransferase 1 (DGAT1) gene (*DGAT1*) is one of the most extensively investigated lipid metabolism genes in livestock. It encodes a microsomal enzyme that catalyzes the terminal and only committed step in triacylglycerol synthesis; thus, it has a key role in the metabolism of cellular glycerolipids [3]. Considerable effort has centered on understanding the effects of *DGAT1* variation on livestock production, with the research focusing on an exon 8 polymorphism (commonly called K232A). Currently, the most notable effect of this polymorphism is on milk production, and for a variety of cattle breeds from different countries, including the Netherlands [4,5], Germany [6,7], France [8], Poland [9], Israel [10], Sweden [11], China [12] and New Zealand [4,13]. 

The effect of the bovine *DGAT1* K232A polymorphism on meat and carcass production has also been widely investigated, but the results obtained lack consistency. Li et al. [14] reported that K232A affected intramuscular fat content (IMF) and marbling in five beef breeds. In Germany, Thaller et al. [6] described a similar effect on IMF, but this effect was only detected in Holstein and not in Charolais cattle. In contrast, Pannier et al. [15] and Aviles et al. [16] reported a lack of association between K232A and IMF in Irish crossbred *Bos taurus* cattle and Spanish crossbred cattle, respectively. With the Spanish crossbred beef cattle, Aviles et al. [15] detected an association between K232A and backfat thickness, and Gill et al. [17] reported K232A to be associated with sirloin weight after maturation and sirloin fat depth in Aberdeen–Angus cross beef cattle. However, Fortes et al. [18] suggested a lack of association of K232A with backfat thickness, ribeye area, and shear force in Brazilian *Bos indicus* and *Bos indicus* × *Bos taurus* beef cattle, and Casas at al. [19] did not detect any effect of K232A on carcass traits, including marbling, fat thickness, tenderness, and longissimus muscle area, in their *Bos indicus* populations.

Sheep *DGAT1*, instead of having the K232A polymorphism, has been reported to contain five single nucleotide polymorphisms (SNPs). These are c.-73C/A in the 5′-unstranslated region, c.191+411C/T and c.191+1265C/T in intron 1, c.279+1415C/T in intron 2, c.897+72C/T in intron 10, and c.1461C/T in exon 17 [20,21]. Previous studies [22,23,24] reported associations between variation in ovine *DGAT1* and some meat/carcass traits (including IMF, marbling, tenderness, backfat thickness, carcass weight, dressing percentage, and fat-tail weight), but these were carried out in Iranian and Chinese indigenous sheep breeds and focused on the exon 17 SNP. There has been only one study investigating the effect of another SNP (c.191+411C/T; rs411875883) and undertaken using a European origin sheep breed: the Texel [20]. Further research in other sheep breeds is needed to validate and increase understanding of these findings.

In this study, we set out to investigate variation in two fragments of ovine *DGAT1* that contained the intron 1 SNP (c.191+411C/T) and the exon 17 SNP (c.1461C/T) in a variety of sheep breeds. We then assessed associations between nucleotide sequence variation in those two regions and carcass traits in Southdown lambs.

## 2. Materials and Methods

### 2.1. Sheep Investigated and Phenotypic Data Collection

A total of 207 sheep in two groups were investigated. The first group was used to ascertain the nature of variation in ovine *DGAT1* and comprised 120 sheep selected from a variety of common breeds and composites in New Zealand (NZ), including NZ Romney, Coopworth, Perendale, Corriedale, Merino, Texel, Suffolk, Southdown, Poll Dorset, and Borderdale. These sheep were sourced from 20 different farms in NZ. The second group consisted of 87 Southdown lambs that were produced by 15 Southdown sires using artificial insemination in ovulation-synchronized Southdown ewes, which were not the ewes in the first group studied. These lambs were born and raised in the same environment and with the same nutrition on a single farm in South Canterbury, NZ, over 2017–2018. They were slaughtered at approximately 120 days of age in late January 2018 at an average weight of 44.8 ± 4.86 (mean ± standard deviation) kg.

Carcass data for the Southdown lambs from the second group were collected from a meat-processing plant. Hot carcass weights (HCW) were measured on the processing chain. They are the weight of the carcass minus the pelt, head, and gut. VIASCAN analysis (Sastek, Australia [25]) was used to estimate the following traits: lean meat yield (expressed as a percentage of HCW) in the loin, leg, and shoulder, and the total lean meat yield (the sum of the leg, loin, and shoulder yields for any given carcass). The width and the depth of the *longissimus dorsi* (eye) muscle was measured at the 12th rib, along with the subcutaneous fat depth in that region.

A blood sample from each sheep was collected by piercing the animal’s ear then placed onto Munktell TFN paper (Munktell Filter AB, Falun, Sweden) and air dried. Genomic DNA was purified from a dried blood spot using a two-step washing procedure [26].

### 2.2. PCR-SSCP Analyses

Two pairs of PCR primers (Table 1) were designed to amplify two fragments of ovine *DGAT1*, based on a published ovine *DGAT1* sequence (GenBank EU178818). One fragment was in intron 1 and covered a previously reported SNP c.191+411C/T [20], while the other fragment spanned exon 17, in which a coding-sequence SNP has been identified [21].

The PCR amplifications were undertaken using 15 µL reactions that contained the DNA on a 1.2 mm punch of the TFN paper, 150 µM of each dNTP (Bioline, London, UK), 3.0 mM Mg^2+^, 0.5 U of Taq DNA polymerase (Qiagen, Hilden, Germany), and 1 × reaction buffer supplied with the enzyme. For the two regions amplified, the reactions contained 0.25 µM of one or the other of the primer pairs. The amplifications were undertaken in Bio-Rad S1000 thermal cyclers (Bio-Rad, Hercules, CA, USA), and the thermal profile included an initial denaturation step at 94 °C for 4 min, followed by 35 cycles of 30 s at 94 °C, 30 s at 64 °C, and 30 s at 72 °C, with a final extension step of 5 min at 72 °C.

Following amplification, a 0.7 µL aliquot of the PCR products was mixed with 7 µL of loading dye (98% formamide, 10 mM EDTA, 0.025% bromophenol blue, 0.025% xylene cyanol). The samples were denatured at 95 °C for 5 min followed by rapid cooling on wet ice. They were then loaded into 16 cm × 18 cm acrylamide:bisacrylamide (37.5:1) (Bio-Rad) gels and electrophoresed with 0.5× TBE buffer using Protean II xi cells (Bio-Rad) and the conditions described in Table 1. The gels were visualized by silver staining using the method of Byun et al. [27].

### 2.3. DNA Sequencing and Sequence Analyses

PCR amplicons representing different SSCP patterns were selected for DNA sequencing in both directions and using the matched PCR primers at the Lincoln University DNA Sequencing Facility, NZ. Amplicons with simple SSCP banding patterns (and hence that appeared to be homozygous) were purified and then directly sequenced. For those rarer SSCP patterns that only appeared to be found in heterozygote forms, a sequencing approach described in Gong et al. [28] was used. In this approach, single bands of interest from the PCR-SSCP gels for heterozygous animals were recovered directly from the gels as a thin slice. This slice was macerated, and the DNA was eluted into 50 µL TE buffer by incubating at 70 °C for 20 min. The original primers and 1 µL of the eluted solution (as a template) were used for a second round of PCR amplification to produce a simple SSCP pattern that could be directly compared to the pattern derived from the original heterogeneous amplicons. The second “homozygous” PCR amplicons were then sequenced in both directions at the Lincoln University DNA Sequencing Facility.

Sequence alignments and translations were undertaken using DNAMAN version 5.2.10 (Lynnon BioSoft, Vaudreuil, QC, Canada).

### 2.4. Statistical Analyses

The statistical analyses were undertaken using Minitab. General linear mixed models were firstly used to test associations between the presence or absence of individual variants of *DGAT1* and variation in carcass traits. For those variants that had associations with *p* < 0.20 and which could thus potentially impact the trait, a second set of models was run with these variants factored in as explanatory elements. This enabled determination of the independent effect of each variant. In all the models, gender and birth rank were fitted as fixed factors, and sire was fitted as a random factor. The model(s) were: Y_ijklm_ = µ + G_i_ + V1_j_ + V2_k_ + Br_l_ + S_m_ + e_ijkl_; where Y_ijklm_ is the trait marginal mean, µ is the group raw mean for the trait, G_i_ is the effect of gender, V1_j_ is the effect of the j^th^ variant (presence and absence), V2_k_ is effect of the second variant presence/absence (if factored in), Br_l_ is the effect of first birth rank, S_m_ is the effect of the m^th^ sire and, e_ijklm_ is the random residual effect. Unless otherwise indicated, all *p* values were considered statistically significant when *p* < 0.05, and trends were noted at *p* < 0.10.

## 3. Results

### 3.1. Variation in Ovine DGAT1

**Figure 1:**
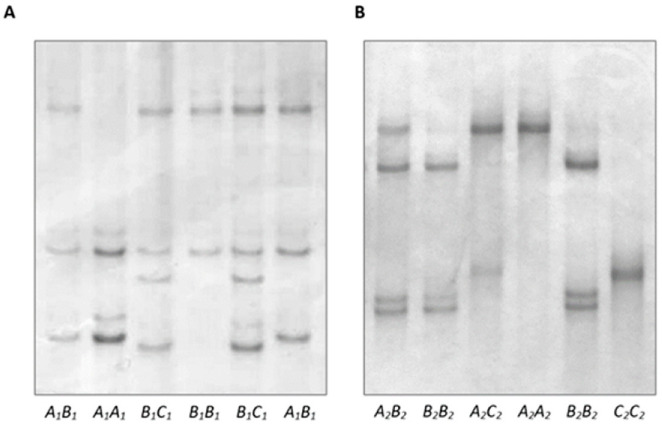
Banding patterns for PCR-SSCP analysis of a fragment of intron 1 (**A**) and a fragment containing exon 17 (**B**) of *DGAT1*. The homozygous genotypes and some heterozygous genotypes for each region are shown.

**Figure 2:**
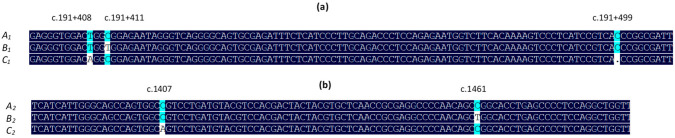
Nucleotide sequence comparisons of the ovine *DGAT1* variants. Three variants in intron 1 (**a**) and three variants in exon 17 (**b**) were detected. Only the nucleotides in the variable region are shown. Nucleotides with high levels of homology are colored, with black indicating 100% homology and blue indicating ≥50%. Positions of nucleotide differences are indicated.

PCR-SSCP analysis of intron 1 amplicons revealed three different banding patterns (Figure 1A), and DNA sequencing of the amplicons revealed three unique nucleotide sequences representing three variants, named *A*_1_, *B*_1,_ and *C*_1_ (Figure 2). The sequence of *A*_1_ was identical to the published ovine *DGAT1* sequence EU178818, and two SNPs (c.191+408T/A and c.191+411C/T) and one insertion/deletion (c.191+499delC) were identified in the other two sequences. The variants occurred at frequencies of 70.8%, 23.3%, and 5.9% for *A*_1_, *B*_1_, and *C*_1_, respectively, in the group of sheep used for variation screening. The sequences of the variants were deposited into GenBank with accession numbers OP235451–OP235453. In the 87 Southdown lambs studied for the association analyses, the variant frequencies were 59.2%, 31.6%, and 9.2% for *A*_1_, *B*_1_, and *C*_1_, respectively, and the genotype frequencies were 28.7% (*n* = 25), 44.8% (*n* = 39), 16.1% (*n* = 14), 8.1% (*n* = 7), and 2.3% (*n* = 2) for *A*_1_*A*_1_, *A*_1_*B*_1_, *A*_1_*C*_1_, *B*_1_*B*_1_, and *B*_1_*C*_1_, respectively.

For exon 17 amplicons, three PCR-SSCP patterns (Figure 1B) representing three sequence variants named *A*_2_, *B*_2,_ and *C*_2_ (Figure 2) were also detected. There were two SNPs (c.1407C/A and c.1461C/T) identified in exon 17, and both SNPs were synonymous. The sequence of variant *A*_2_ was identical to the published ovine *DGAT1* sequence EU178818, and the sequences of all these variants were deposited into GenBank with accession numbers OP235454–OP235456. In the sheep that were used for variation screening, *A*_2_ was most common (at a frequency of 89.1%), whereas *B*_2_ and *C*_2_ were much less common, with frequencies of 9.2% and 1.7%, respectively. Because of the low frequencies, the association analyses were only carried out for variation in intron 1, but not the exon 17 region.

### 3.2. Association of DGAT1 Intron 1 Variation and Carcass Traits

The presence of the *B*_1_ variant was found to be associated with an increase in loin yield, shoulder yield, and leg yield in the single-variant model (Table 2). The association with loin yield persisted in the second set of models, whereas there was only a trend towards significance for shoulder and leg yields in these models (Table 2). A trend towards association was observed for variant *C*_1_ and shoulder yield in the single-variant model, but this association was lost in the multi-variant model (Table 2).

## 4. Discussion

This study investigated sequence variation in intron 1 and exon 17 regions of ovine *DGAT1* and describes an association with *longissimus dorsi* meat yield in NZ Southdown lambs. Three nucleotide sequences were detected in each region, and the sequence variation included two previously reported SNPs, two new SNPs, and a previously unidentified nucleotide deletion. The SNP density observed was higher than the average of 4.9 SNPs per kb across the sheep genome suggested by Kijas et al. [29], and, perhaps unsurprisingly, the intron 1 region appeared to be more variable than the exon 17 region.

The detection of an intron 1 variant associated with increased loin meat yield suggests that variation in the gene has a role to play in how this muscle tissue is accreted. As the association was detected for variant *B*_1_ but not for *A*_1_, and these variants only differed by the SNP c.191+411C/T, it seems unlikely that the association detected is due directly to the effect of this SNP. However, this SNP may be linked to other functional SNPs outside the region investigated here or be part of a hitherto unrecognized region that in some way affects gene expression. In this respect, in most species of animals and plants, the first intron is the longest and typically the most conserved. It can have a higher density of regulatory elements or functional motifs compared to other downstream introns, and accumulating evidence suggests some direct functional roles for the first intron, including the regulation of mRNA splicing, or nonsense-mediated decay [30,31].

The effect of the intron 1 SNP c.191+411C/T was investigated previously in 255 Texel lambs by Armstrong et al. [20], but the effects they described are different to those reported here. These authors reported associations of this SNP with hot carcass weight, shoulder weight, ribeye area, and fat thickness, with TT (corresponding to *B*_1_ in this study) animals showing a decrease in the values while CC (corresponding to *A*_1_ and *C*_1_ in this study) animals showed an increase. These associations were either not detected with the Southdown sheep or possibly, in the case of ribeye area, slightly at odds with the associations described here. While the *B*_1_ variant was associated with loin meat yield, no effect on either eye muscle width or depth was observed, suggesting that the effect, if any, was a result of variation in muscle length or shape (the muscle is ovoid).

While this is speculative, there were other differences in the sheep investigated. For example, the Texel lambs described by Armstrong et al. [20] appear to be different in respect to both weight and fatness. They had a lower mean HCW (16.89 kg) but with a higher standard deviation (3.88 kg), compared to the mean of 20.05 kg and standard deviation of 2.33 kg for the Southdown lambs. In this respect, the Texel lambs were slaughtered at 291 ± 22 days of age and had a slaughter weight of 36.8 ± 5.3 (mean ± standard deviation) kg (versus 44.8 ± 4.86 kg in this study). The Texel lambs were leaner and had a fat thickness of 2.64 ± 0.76 (mean ± standard deviation) mm, whereas the Southdown lambs had fat thickness of 4.15 ± 1.08 (mean ± standard deviation) mm. This suggests that the Southdown lamb carcasses, which were “finished” for slaughter on mixed ryegrass/clover/chicory pasture, were far more uniform, and this uniformity may have masked any genetic effect of *DGAT1* on the measured carcass traits.

The carcass traits were also only measured in male lambs for the Texel sheep [20], whereas in our study, the traits were measured for both male and female lambs. Together, this suggests that the genetic effects detected may be influenced by other factors, including gender, growth/body condition, and breed, and noting that gender effects were corrected for in our models.

In Iranian and Chinese indigenous sheep breeds, Ala Noshahr and Rafat [22], Mohammadi et al. [23], and Xu et al. [24] reported that the common allele (with a frequency ranging from 59% to 83%) was T (corresponding to the *B*_2_ variant reported here) for the exon 17 SNP c.1461C/T. They described associations between this SNP and various carcass and meat traits. However, in our study, we found that allele C (corresponding to variants *A*_2_ and *C*_2_) was most common, whereas the T allele equivalent occurred at an overall frequency of only 9.2% in the NZ sheep from a variety of breeds and composites of differing breeds. The sheep genome assembly sequence (NC_056062.1) also has allele C at this SNP. It is unknown whether this difference in allele frequencies reflects genetic difference in breeds or is the consequence of selection for production traits, and this requires further investigation.

## 5. Conclusions

In this study, we analyzed sequence variation in intron 1 and exon 17 of ovine *DGAT1* and report three sequence variants for each region. An intron 1 variant (*B*_1_) was found to be associated with increased loin meat yield in NZ Southdown sheep. The effect of variation in exon 17 was not investigated, but the frequencies of variants were notably different to those reported in other sheep breeds.

## Figures and Tables

**Table 1:** PCR primers and SSCP analysis conditions for the two *DGAT1* regions.

Region Amplified	Primer Sequence (5′-3′)	Expected Size (bp)	SSCP Condition
Intron 1	GTGATCCCACCACGCACAG	271	10% gel containing 1% glycerol, 150 V, 24 °C and 17 h.
	CACTTTGACCCTAGAGCAG		
Exon 17	TGATGGCACAGGTGAGCAG	310	12% gel, 250 V, 22 °C and 17 h.
	CAGGCTCCAGTACAGCAGC		

**Table 2:** Association between the presence or absence of *DGAT1* intron 1 variants and carcass traits.

Trait	Variant Assessed	Other Variant Fitted	Mean ± SE		*p* ^1^
			Present	Absent	
HCW (kg)	*A*_1_	None	19.9 ± 0.37 (*n* = 78)	19.6 ± 0.94 (*n* = 9)	0.766
	*B*_1_	None	20.0 ± 0.46 (*n* = 48)	19.8 ± 0.48 (*n* = 39)	0.662
	*C*_1_	None	19.6 ± 0.78 (*n* = 16)	20.0 ± 0.39 (*n* = 71)	0.639
Loin yield (%)	*A*_1_	None	15.5 ± 0.09 (*n* = 78)	15.8 ± 0.24 (*n* = 9)	0.196
	*B*_1_	None	15.8 ± 0.11 (*n* = 48)	15.3 ± 0.11 (*n* = 39)	**0.001**
	*C*_1_	None	15.3 ± 0.19 (*n* = 16)	15.6 ± 0.10 (*n* = 71)	0.200
	*B*_1_	*A*_1_	15.8 ± 0.14 (*n* = 48)	15.4 ± 0.16 (*n* = 39)	**0.003**
Shoulder yield (%)	*A*_1_	None	16.9 ± 0.11 (*n* = 78)	17.1 ± 0.29 (*n* = 9)	0.424
	*B*_1_	None	17.1 ± 0.14 (*n* = 48)	16.7 ± 0.14 (*n* = 39)	**0.014**
	*C*_1_	None	16.5 ± 0.23 (*n* = 16)	17.0 ± 0.12 (*n* = 71)	*0.080*
	*B*_1_	*C*_1_	17.0 ± 0.20 (*n* = 48)	16.7 ± 0.15 (*n* = 39)	*0.065*
	*C*_1_	*B*_1_	16.7 ± 0.25 (*n* = 16)	16.9 ± 0.12 (*n* = 71)	0.501
Leg yield (%)	*A*_1_	None	22.3 ± 0.13 (*n* = 78)	22.8 ± 0.34 (*n* = 9)	0.168
	*B*_1_	None	22.6 ± 0.16 (*n* = 48)	22.1 ± 0.17 (*n* = 39)	**0.037**
	*C*_1_	None	22.2 ± 0.28 (*n* = 16)	22.4 ± 0.14 (*n* = 71)	0.460
	*B*_1_	*A*_1_	22.7 ± 0.20 (*n* = 48)	22.3 ± 0.24 (*n* = 39)	*0.073*
Eye muscle width (mm)	*A*_1_	None	68.7 ± 0.63 (*n* = 78)	67.6 ± 1.58 (*n* = 9)	0.485
	*B*_1_	None	68.8 ± 0.78 (*n* = 48)	68.4 ± 0.81 (*n* = 39)	0.734
	*C*_1_	None	68.4 ± 1.31 (*n* = 16)	68.6 ± 0.65 (*n* = 71)	0.850
Eye muscle depth (mm)	*A*_1_	None	29.2 ± 0.29 (*n* = 78)	29.1 ± 0.74 (*n* = 9)	0.878
	*B*_1_	None	29.3 ± 0.36 (*n* = 48)	29.2 ± 0.38 (*n* = 39)	0.817
	*C*_1_	None	29.5 ± 0.61 (*n* = 16)	29.2 ± 0.30 (*n* = 71)	0.661
IMF (%)	*A*_1_	None	3.32 ± 0.135 (*n* = 78)	2.94 ± 0.330 (*n* = 9)	0.252
	*B*_1_	None	3.35 ± 0.172 (*n* = 48)	3.23 ± 0.168 (*n* = 39)	0.554
	*C*_1_	None	3.18 ± 0.280 (*n* = 16)	3.31 ± 0.141 (*n* = 71)	0.664
Subcutaneous fat depth (mm)	*A*_1_	None	4.12 ± 0.193 (*n* = 78)	4.25 ± 0.557 (*n* = 9)	0.830
	*B*_1_	None	4.18 ± 0.240 (*n* = 48)	4.08 ± 0.255 (*n* = 39)	0.767
	*C*_1_	None	3.79 ± 0.438 (*n* = 16)	4.21 ± 0.206 (*n* = 71)	0.397

^1^*p* < 0.05 in bold, and *p* < 0.10 in italic.

## Data Availability

The original data used in this paper are available by contacting the corresponding author upon request.
